# Ovarian Fibroma Presents As Uterine Leiomyoma in a 61-Year-Old Female: A Case Study

**DOI:** 10.7759/cureus.36264

**Published:** 2023-03-16

**Authors:** Emmanuella Borukh, Benjamin Ilyaev, Sabina N Muminiy, Matthew Babayev, Yakubmiyer Musheyev, Maria Levada

**Affiliations:** 1 Medicine, Yeshiva University, New York City, USA; 2 Medicine, Hofstra University, Hempstead, USA; 3 Medicine, St. Francis College, New York City, USA; 4 Medicine, Sophie Davis Biomedical Education Program at the City University of New York (CUNY) School of Medicine, New York City, USA; 5 Medicine, New York Institute of Technology College of Osteopathic Medicine (NYITCOM), Old Westbury, USA; 6 Obstetrics and Gynecology, New York Institute of Technology College of Osteopathic Medicine (NYITCOM), Old Westbury, USA

**Keywords:** computed tomography (ct), magnetic-resonance imaging (mri), ultrasound sonography, torsion, ovarian cyst, uterine fibroids, uterine leiomyoma, ovarian fibroma

## Abstract

Uterine leiomyoma should be considered when a female patient reports symptoms of abdominal pressure and abnormal vaginal bleeding. However, the symptoms of a uterine leiomyoma are vast and overlap with other possible diseases that are difficult to distinguish even with imaging studies. This is why it is important for physicians and healthcare providers to keep an open mind and have a broad differential diagnosis.

In this case study, we present a 61-year-old postmenopausal female patient who presented to the emergency department with complaints of pelvic and abdominal pain, as well as vomiting and diarrhea. She was admitted for observation. A complete blood count (CBC), comprehensive metabolic panel (CMP), and urinalysis revealed no abnormalities; a pelvic ultrasound and CT scan reported possible adnexal torsion. The patient remained stable and the pain had subsided when she was seen the next morning by her gynecologist (GYN) who discharged her to follow-up in the office. Subsequent examinations that aided in the diagnosis included, but were not limited to pelvic and transvaginal ultrasounds, an abdominal and pelvic CT, and a pelvic MRI. In this case, the MRI revealed an 11-cm mass that could represent a torsioned pedunculated necrotic fibroid originating from the uterus. Radiology recommended surgical removal. Upon removal and review of the pathology of the mass, it was revealed to be a torsioned, partially necrotic fibroma that had originated from the ovary and not from the uterus, as imaging had originally suggested.

## Introduction

Ovarian fibromas are solid tumors that appear in ovaries, usually of benign histology (neoplasm), that are generally diagnosed in premenopausal and menopausal women [1]. Ovarian fibromas occurrence rate accounts for 1-4% of all ovarian tumors [1]. One percent of these cases present as Meigs syndrome, a triad consisting of a benign ovarian tumor accompanied by pleural effusion and ascites [2,3]. Typically, Meigs syndrome is developed in postmenopausal women [3]. Ovarian tumors in young women and teenagers are associated with Gorlin’s syndrome, a rare autosomal dominant genetic disorder that can affect various organs of the body, including the ovaries [4,5]. The syndrome is versatile in its phenotypic expressions and may manifest as basal cell carcinomas, odontogenic keratocysts, palmoplantar pits, skeletal abnormalities as well as various tumors which include ovarian fibromas which may present as both unilateral and bilateral [5].

Ovarian fibromas are usually asymptomatic and grow until detected during gynecological examination; in a retrospective study of 23 patients with ovarian fibromas, the average size was 14 cm [6]. Sometimes patients may notice abdominal enlargement and urinary problems, and experience heaviness, discomfort, and pain [1]. If left undetected, they may grow up to occupy the whole abdominal cavity causing severe complications [7]. It is often difficult to diagnose ovarian fibroma due to its similarity with uterine tumors [1,8]. It has been reported that 34% of ovarian fibromas are misdiagnosed for uterine leiomyomas primarily due to the similarities in symptoms, pathology, and appearance on ultrasound (US) images as solid masses [1,8]. However, unlike ovarian fibromas, uterine leiomyomas can appear in 50% of all women of reproductive age [9]. Magnetic resonance imaging (MRI) and/or computerized tomography (CT) scan procedures are also considered for more precise characterization and differentiation from other tumors if any are present [1]. Although ultrasound has been one of the accessible and cost-effective instruments for diagnosing pelvic masses, it may still be inadequate for proper diagnosis [10]. In particular, ovarian fibromas typically appear on ultrasound as solid, hypoechoic masses with attenuation of the ultrasound beam [11]. They may therefore resemble a pedunculated subserosal uterine fibroid in appearance, leading to further misdiagnosis [11].

The differentiation of ovarian masses from uterine leiomyomas can be accomplished by localizing a mass. If the mass is found to be detached from the ovaries or adjacent to round ligaments, then ovarian etiology is improbable. In addition, the presence of bridging vascular signs, which are essentially vessels that extend from the uterus and supply pelvic mass, is indicative of uterine leiomyoma [11]. In a previous patient study, it has been reported that 20 out of 26 leiomyomas presented with bridging vascular signs while ovarian masses showed no such signs at all [10]. Also, MRI procedures provide morphological characteristics of masses that help distinguish benign tumors from malignant tumors [12]. The cystic mass is usually associated with a benign tumor, but if the solid soft tissue is detected within the mass, it may be identified as benign, borderline malignant, or malignant [12]. The size of the tumor (>10 cm), hypervascularity within the mass, extension of mass into the pelvis, or surrounding viscera can also suggest malignancy [1]. However, the accuracy of the MRI imaging in distinguishing benign from malignant tumors is only 80%; thus, it is safe to say that the true nature of a tumor is determined intraoperatively by performing a frozen section (FS) pathology procedure [13,14].

Occasionally, an ovarian fibroma may be pedunculated resulting in partial or complete twisting/rotation of the mass around its pedicle, restricting the arterial and venous flow [15]. MRI findings suggesting torsion include thickening and hemorrhage of tubes, torsion knot, as well as a deviation to the twisted mass [1]. The most common symptoms of torsion of ovarian fibroma are acute pain in the lower abdomen, some patients may present with nausea and vomiting [16]. The torsion may result in tissue necrosis, inflammation, and peritonitis which is why early diagnosis and treatment are important [17].

Laparoscopy is the standard procedure for dealing with benign adnexal masses that involves less hospital stay, quicker recovery time as well as less postoperative pain [18]. However, as the risk of misdiagnosis of malignant ovarian tumors remains, so does the risk of rupturing the mass and spillage of potentially malignant cells into the system circulation [18]. For patients of postmenopausal age, total hysterectomy or oophorectomy are preferable procedures to reduce the risk of rapture of cystic contents, intraperitoneal dissemination, ovarian and/or breast cancer, and repeated surgery in case of recurrence of tumors [19,18]. However, in younger or pregnant patients, more conservative surgery is considered to preserve the ovary via laparoscopic approach involving resecting the tumor, untwisting the ovary, and restoring proper blood flow [20,21].

## Case presentation

A 61-year-old postmenopausal woman presented to the emergency department with complaints of intense pelvic and abdominal pain radiating to her lower back, vomiting, and diarrhea. The patient also had symptoms of nocturia, recurrent urinary tract infections, frequent urination, and female urinary stress incontinence. She had a family history of breast cancer, which her maternal aunt had in her 50s and her cousin had in her 40s. Her social history showed that she was a former smoker but was unremarkable in regard to exercise, diet, and stress levels. Her past medical history was significant for osteopenia and her surgical history was significant for a right kidney malignant mass removal in 2015 and a kidney stone operation in 1995.

A complete blood count (CBC), comprehensive metabolic panel (CMP), and urinalysis revealed no abnormalities. The patient was admitted for observation by her gynecologist. A transabdominal pelvic/kidney ultrasonogram was negative for stones but revealed uterine fibroids, the largest of which measured 10.1 × 9.7 × 11.5 cm. The patient was feeling better in the morning and CBC, as well as vital signs, were stable. Further, because of a previous ultrasound, done 15 days prior to the emergency room (ER) visit, in which both ovaries were visualized, it was deemed that there was a low risk of ovarian torsion and that surgical intervention was not indicated (Table 1). The patient was discharged and scheduled a follow-up in the office.

**Table 1:** Imaging results and operative diagnosis. US: ultrasound

Date	Imaging modality	Result	Origin of growth
Fifteen days prior to ER visit	US transabdominal and transvaginal pelvic	Pedunculated uterine fibroid noted measuring 9.77 × 7.17 × 11.72 cm. Both ovaries visualized - right ovary measured 1.29 × 0.92 × 1.03 cm, volume 0.64 mL and left ovary measured 1.15 × 0.61 × 0.86 cm, volume 0.31 mL. Bilateral ovarian Doppler demonstrated	Fibroid deemed to come from the uterus
Initial presentation - ER visit	US transabdominal and transvaginal pelvic	Negative for stones but showed uterine fibroids	Fibroid deemed to come from the uterus
Initial presentation - ER visit	CT abdomen and pelvis	11.7 × 10.3 cm low-attenuation right adnexal mass with suggestion of twisted ovarian vascular pedicle. No uterine or left adnexal abnormality	Fibroma deemed to come from the right ovary
Ten days after ER visit	MRI-3T pelvis pre and post IV contrast	Large enhancing mass anterior to the uterine fundus measuring 10.5 x 9.5 x 11.3 cm. Mass could represent pedunculated partially necrotic fibroid, torsed and disconnected from the uterus	Fibroid deemed to come from the uterus
Twenty-five days after ER visit	Operative diagnosis	13 × 12 cm hard/calcified right adnexal mass, ovarian fibroma with partial torsion	Fibroma deemed to come from the right ovary

The differential diagnosis for a postmenopausal patient presenting with abdominal and pelvic pain and suspected uterine fibroid is consistent with a possibly degenerating uterine leiomyoma.

The patient returned to her obstetrician-gynecologist (OB-GYN) a few days after her emergency room visit with complaints of severe pain again in her lower abdomen, right groin, right buttock, and with occasional nausea. The decision was made to further explore and reevaluate the patient’s symptoms through supplementary testing. The patient underwent additional pelvic and transvaginal ultrasound, an abdominal and pelvic CT, and a pelvic MRI with and without intravenous contrast. The MRI findings revealed a solid mass measuring 10.5 × 9.5 × 11.3 cm anterior to the uterine fundus with numerous internal non-enhancing cystic spaces. The mass slightly abuts the anterior uterine fundus but appears to cause mass effect on the uterus. Immediately inferior to this 11 cm mass, a tubular cystic lesion measuring 3.2 × 5.2 × 5.3 cm was found. The uterus was also found to be anteverted, slightly anteflexed, and normal-sized. The findings also included a right ovarian cyst measuring 1.1 cm. The 11 cm solid mass was presumed to represent a pedunculated partially necrotic fibroid that had torsed and disconnected from the uterus. Radiology recommended surgical resection.

**Figure 1:**
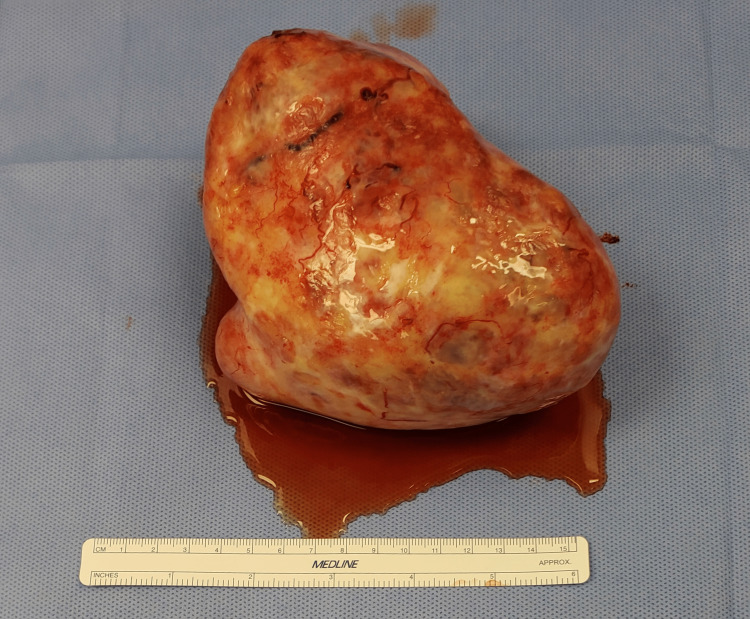
Torsed ovarian fibroma.

**Figure 2:**
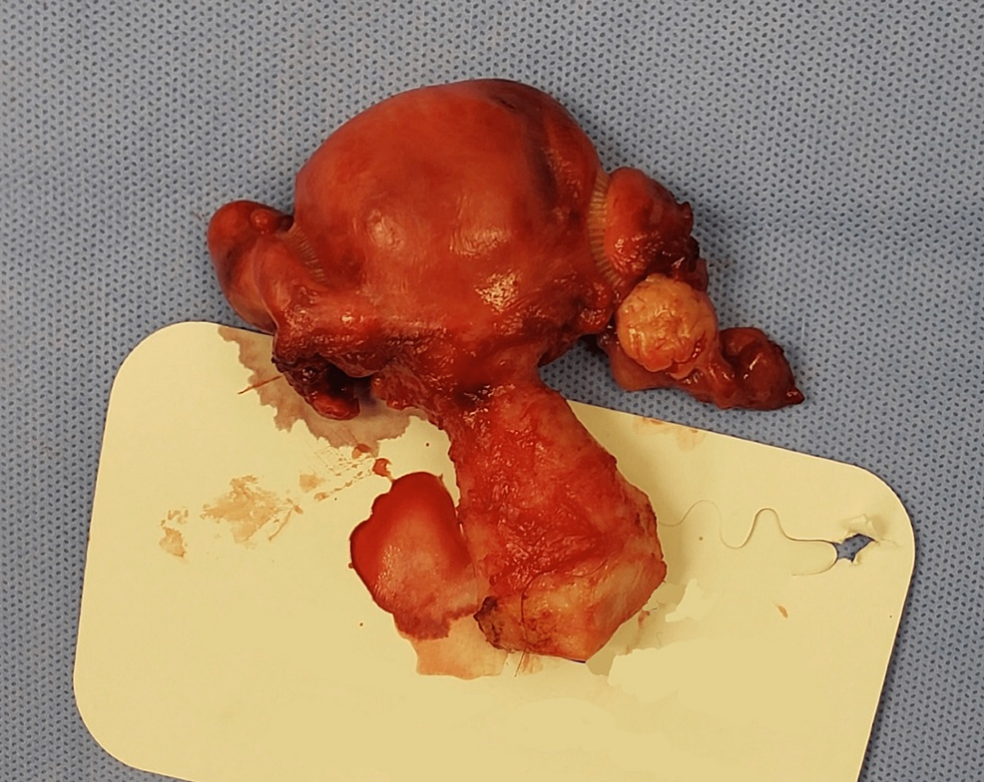
Uterine cervix, left ovary, and both fallopian tubes.

The preoperative diagnosis was uterine leiomyoma. The patient was scheduled for a total abdominal hysterectomy with removal of tubes and ovaries, excision of adnexal mass, uterosacral colposuspension, lysis of ovarian cyst, and an exploratory laparotomy. The procedure was conducted under general anesthesia. Visualization of the pelvic organs showed a partially torsioned right adnexal mass, measuring 14 × 10 × 10 cm with many areas of cystic degeneration and hemorrhage as well as a cyst with two pieces of smooth hemorrhagic friable tissue measuring 2.5 × 2 × 0.5 cm (Figures 1, 2). The right ovary was not observed separate from the mass. According to preoperative imaging, the diagnosis was uterine leiomyoma. However, intraoperative diagnosis confirmed a right ovarian fibroma. Following procedure termination, the patient was brought to the recovery room in stable condition. Specimens sent to cytology included aspiration peritoneal fluid and contents of right ovarian cyst, specimens sent to pathology included right adnexal mass, uterus with left ovary, and bilateral fallopian tubes.

The standard treatment of torsed ovarian fibroma, or any organ torsion, is surgery. Total hysterectomy is generally suggested for postmenopausal patients as there is a risk of recurrence of masses as well as the risk of developing ovarian or breast cancer [19]. In our case, the patient underwent total abdominal hysterectomy with the removal of tubes and ovaries and excision of adnexal mass. After the procedure, the patient’s hospital course was uneventful and a follow-up appointment was scheduled a week following the surgery. Cytology and pathology reports were negative for malignancy.

## Discussion

The symptoms of a large torsioned ovarian fibroma are very similar to those of malignant ovarian tumors and of degenerating uterine leiomyomas [8]. Symptoms usually have a very sudden onset including sharp abdominal pain and discomfort accompanied by nausea and vomiting [6]. In order to diagnose ovarian fibroma, the clinician may use various methods of medical imaging including ultrasound (US), computed tomography (CT), and magnetic resonance imaging (MRI). These imaging techniques differ in their accuracy in locating the precise origin of a fibroma, so often exploratory surgery is needed to confirm the predicted diagnosis.

In the case presented above, the patient was believed to have a degenerating pedunculated uterine leiomyoma. This was concluded due to the patient's symptoms, past medical history, and medical imaging, specifically the MRI, US, and CT scan. However, surgical intervention confirmed the diagnosis to be a torsed right ovarian fibroma. It is interesting to note that there have been other medical situations documented where medical imaging caused the diagnosis to be overlooked. A similar case report discussed the repercussions of overlooking the diagnosis of a Qatari female, who reported to her obstetrician-gynecologist (OB-GYN) with symptoms of minimal vaginal bleeding for five days [22]. A pelvic ultrasound, Doppler ultrasound, MRI, and a colonoscopy was done prior to surgical intervention [22]. The preoperative provisional diagnosis, based on these scans, was thought to be an adnexal mass. However, upon surgical incision, the intraoperative diagnosis proved to be a tumor appendicular in origin [22]. Only after the surgeon visualized the tumor, the intraoperative diagnosis was definitively concluded [22].

As with most cases involving abdominal pain, ultrasonography imaging is the initial emergent examination for diagnosis [23] but oftentimes is not always reliable in predicting the origin of these tumors [8]. In the identification of solid tissues in adnexal masses, transvaginal US had a sensitivity, specificity, and accuracy of 97%, 46%, and 68%, respectively [24]. US also demonstrates numerous false-positive results, especially when evaluating lesions adherent to the uterine wall, such as blood or fatty tissue [24]. Ultrasonography has had its limitations in the detection of other diseases as well. Transvaginal sonography could detect submucous fibroids, a fibroid developing in the inner layer of the uterus, but it is difficult to differentiate this from polyps [10]. Additionally, the accuracy of sonography depends significantly on uterine volume and the precise number and position of the myomas [10]. The larger an ovarian fibroma is, the harder it becomes to diagnose, because a larger ovarian fibroma mimics a uterine leiomyoma and sometimes even a malignant ovarian tumor. Thus, such situations require further evaluation with MRI.

MRI is widely used in the diagnosis of gynecological disorders because it depicts a higher resolution relative to CT and ultrasound [25]. In testing the validity of MRI and ultrasound in the prediction of uterine fibroids, MRI was consistently more efficient with 97.1% sensitivity, 83.3% specificity, and 95.0% accuracy [26]. The greater specificity of MRI is due to its ability to correctly identify dermoid, endometriotic cysts, and fibroids - which may appear malignant in ultrasound [25]. Therefore, evidence has been in favor of using MRI as the primary diagnostic tool for patients with adnexal masses in the uterine area. Although studies cite MRI to have high resolution and specificity [25], CT scan is still a viable technique in the pretreatment evaluation of ovarian cancer to define the extent of disease and assess the likelihood of optimal surgical cytoreduction [27].

In this case, MRI incorrectly identified the fibroma as originating from the uterus instead of the ovary, whereas additional CT scan suggested an ovarian fibroma, which was confirmed in surgery. Therefore, CT scans are also accurate in the diagnosis of the origin of ovarian fibromas. CT is preferred over MRI because it is often more available and quicker. MRI and CT share a similar staging accuracy of 70-90%, but given MRI’s high cost and relatively long examination times, CT is the recommended imaging modality of choice for staging ovarian cancer [25]. CT is effective in identifying a well-defined adnexal mass with a smooth border, which is indicative of a twisted pedicle and thus an adnexal torsion [28,29]. CT has been shown to predict suboptimal cytoreduction with sensitivity of 79% and specificity of 75% [27]. To improve detection rates for these masses, a coronal reformation can be added to a transverse CT which greatly improves the detection rate of this finding from 27-29% to 77-79% [29]. Twisting of the adnexal pedicle had the highest accuracy (75-80%) among all CT findings and then adding a coronal reformation significantly increased overall accuracy (87-92%) and sensitivity (87%) for detecting adnexal torsion [29].

Although this case did not use a coronal reformation, CT was still able to detect a twisted pedicle and consequently accurately diagnosed the mass as an adnexal torsion originating from the right ovary. It is evident that the accuracy of these scanning methodologies varies, and MRI and CT are both useful while the results of the ultrasound are often inconclusive and ambiguous. A combination of initial ultrasound and further MRI and CT may be used for the diagnosis of ovarian fibromas but, because of the varying accuracy of these scans, only after exploratory surgery can a definitive conclusion be made.

## Conclusions

This study described the symptoms, pre and postoperative diagnosis, and the various treatments correlated with ovarian fibromas. It specifically discusses a female who presented to the emergency department with what appeared to be symptoms of a degenerating uterine leiomyoma but ultimately ended up being diagnosed with a torsioned ovarian fibroma. In order to avoid adverse outcomes which can lead to further complications, it is important for physicians to consider various differential diagnoses in order to properly treat the patient for any and all ailments the patient may present with. While surgery is not used as an initial diagnostic method, it is clear that imaging only provides a certain level of accuracy. Due to these limitations, only after surgery can a definitively accurate diagnosis be established.
